# Differential resting-state MEG microstate patterns in migraineurs: a cross-sectional study

**DOI:** 10.1186/s10194-025-02168-z

**Published:** 2025-10-14

**Authors:** Peiqi He, Changling Li, Wei Sun, Ning Chen, Mengmeng Ma, Jinghuan Fang, Shiqin Li, Hui Lang, Haoyang Xing, Li He

**Affiliations:** 1 Department of Neurology, West China Hospital of Sichuan University, Chengdu, Sichuan China; 2 Institute of Radiology and Medical Imaging, Department of Radiology, West China Hospital of Sichuan University, Chengdu, Sichuan China; 3 College of Physics, Sichuan University, Chengdu, Sichuan China; 4 West China Hospital of Sichuan University, No. 37, Wainan Guoxue Xiang, Chengdu, China

**Keywords:** Migraine, MEG microstate, Resting-state, OPM-MEG

## Abstract

**Background:**

Magnetoencephalography (MEG) microstates offer distinct and complementary insights into brain activity compared to electroencephalography microstates, but their role in migraine remains incompletely understood. This study used optically pumped magnetometer MEG (OPM-MEG) to compare resting-state microstate patterns between migraineurs and healthy controls and to evaluate their correlations with clinical characteristics.

**Methods:**

Resting-state OPM-MEG data were collected in this cross-sectional study. Parameters of six MEG microstate classes (Ms1-Ms6) were calculated and compared between migraineurs and healthy controls. The E-value and its lower control limit were assessed for sensitivity analysis. Pearson correlation coefficients were employed to investigate the relationships between microstate parameters and clinical traits.

**Results:**

The study included 25 migraine patients without aura (21 females) and 18 healthy controls (13 females). Compared to healthy controls, migraineurs exhibited significantly higher coverage and occurrence per second of Ms2 and greater coverage of Ms6, whereas these parameters of Ms3 were lower. Transition probabilities among microstates (especially Ms2, Ms3, and Ms6) also differed between groups. In migraineurs, the coverage and transition probabilities of Ms3 and Ms6 were negatively correlated with headache attack duration, intensity, and scores on the Headache Impact Test-6.

**Conclusions:**

These findings provided evidence for a pathological basis of the synergistic dysfunction across brain networks in migraineurs. OPM-MEG microstate parameters might serve as potential biomarkers for migraine diagnosis and treatment.

**Supplementary Information:**

The online version contains supplementary material available at 10.1186/s10194-025-02168-z.

## Introduction

Migraine is a complex functional brain disorder, characterized by recurrent moderate to severe headaches, often with sensitivity to sound and light, mood changes, and cognitive dysfunction [1–3]. It affects approximately 14% of the global population and is the leading cause of disability in women aged 16 to 49 years [4, 5]. Traditionally, migraine research has relied on functional magnetic resonance imaging (fMRI) and electroencephalogram (EEG) to investigate brain function changes. However, advanced magnetoencephalography (MEG) technology now offers better insights into migraine pathophysiology and biomarker identification [6].

Microstates, defined as quasi-stable scalp potential distributions lasting 40-120ms, reflect the rapid dynamics of large-scale brain networks [7, 8]. Microstate analysis could identify neurophysiological markers for psychiatric and neurological disorders [9]. Previous studies on EEG microstates revealed significant differences in parameters like time coverage, duration, occurrence, and syntax between migraineurs and healthy controls, as well as among different migraine subtypes and phases. These parameters also significantly correlated with headache characteristics and clinical assessments of disability [10–13], offering insights into the pathological mechanisms affecting brain networks in migraine. While EEG and MEG microstates share some similarities, MEG microstates provide unique insights into brain activity [14, 15]. However, the spatiotemporal dynamics of MEG microstates in migraineurs and their relationships with clinical features remain incompletely understood.

Over the past decade, optically pumped magnetometer MEG (OPM-MEG) has significantly advanced non-invasive brain imaging, offering improved sensitivity, spatial resolution, motion robustness, and reduced system complexity [16]. A 64-channel OPM-MEG system matches the performance of a 306-channel Superconducting Quantum Interference Device MEG (SQUID-MEG) system, while a 128-channel OPM-MEG system surpasses it [17]. OPM-MEG can also capture activity from deep brain regions, such as the cerebellum and hippocampus, which are challenging for EEG and SQUID-MEG [18]. Recent research confirms the reliability of resting-state microstates from full-head OPM-MEG recordings [19].

Consequently, this study used OPM-MEG and microstate analysis to compare resting-state microstates between migraineurs and healthy controls, examining their correlations with clinical characteristics. This approach aimed to enhance the understanding of pathophysiological mechanisms underlying migraine.

## Methods

### Participants

This cross-sectional study recruited patients with migraine without aura, as defined by the International Classification of Headache Disorders, 3rd edition (ICHD-III) [20], from the tertiary headache clinic at West China Hospital between January 2024 and January 2025. Diagnoses were confirmed by a minimum of two headache specialists, and patients underwent MEG/MRI scans during the interictal phase, defined by at least three days without migraine attacks before and after the scans [21]. The inclusion criteria for migraineurs were as follows: age between 18 and 60 years, right-handedness, a headache attack frequency of fewer than 15 days per month, and no use of prophylactic medications in the preceding three months. The exclusion criteria included: (1) the presence of other chronic pain disorders, systemic diseases, or significant neurological or psychiatric disorders; (2) a current or past history of analgesic overuse; and (3) contraindications for MRI or MEG scanning.

A control group of healthy, right-handed subjects, matched by sex and age, was recruited and evaluated by neurologists to ensure they had no history of systemic diseases, primary headaches, chronic pain, or neurological/psychiatric disorders, and normal neurological examinations.

The cross-sectional study received approval from the Ethics Committee of West China Hospital, Sichuan University (No. 2024 − 864), and informed consent was obtained from all participants.

### Baseline clinical data collection

Demographic data of all participants and clinical characteristics of migraineurs were collected. The information included basic details such as age, sex, and educational level, along with migraine-related metrics like duration of migraine, frequency and duration of headache attacks, and headache intensity (measured using a Visual Analogue Scale [VAS] ranging from 0 to 10). Data on accompanied symptoms and the locus of headache were also documented. Additionally, the following assessments were recorded: the Migraine Disability Assessment Score (MIDAS), the Headache Impact Test-6 (HIT-6), the 14-item Hamilton Anxiety Scale (HAMA-14), and the 24-item Hamilton Depression Scale (HAMD-24).

### OPM-MEG and structural MRI data acquisition

MEG scanning sessions were conducted in the afternoon from 2:00 to 5:00 p.m., with participants abstaining from sleep deprivation and alcohol the day before. All participants underwent a single MEG scan during the interictal phase using a PyraMag Epoch OPM-MEG system (Beijing Quanmag Healthcare Co. Ltd., Beijing, China). This system primarily comprises a scanning bed, a magnetically shielded cylinder, an acquisition array with 64 single-axis magnetometers, and an associated control system [22]. The cylindrical shield created a shielded region measuring 25 × 25 × 25 cm^3^, with the residual magnetic field and its gradient around the participant’s head measuring approximately 0.3 nT and 0.3 nT/m, respectively, within the shielded area [19]. The OPM sensors demonstrated a noise floor approximately 20 fT/√ Hz and a bandwidth exceeding 100 Hz. These sensors were evenly distributed around the scalp using a 3D-printed helmet designed for full-head coverage [19]. Each participant collected full-head MEG data for a duration of 8 min while remaining quiet with their eyes closed in a dark room. The raw OPM-MEG data were recorded at a sampling rate of 1000 Hz using a 24-bit digital acquisition system.

Following the MEG recordings, participant’s 3D T1-weighted cerebral MRI data were acquired using a 3-Tesla MRI scanner (Siemens Trio Tim, Erlangen, Germany, TR = 7600 ms, TE = 3.0 ms) with a 1 mm isotropic voxel resolution at the Department of Radiology, West China Hospital of Sichuan University. Participants were instructed to remain still, keep their eyes closed, stay awake, and allow their minds to wander. Structural images of all participants were examined by two experienced radiologists, and individuals with any incidental findings on the MRI images were excluded from the study.

### MEG data preprocessing

Data preprocessing was conducted using the Brainstorm software in MATLAB (R2022b). Resting-state MEG data were filtered using a bandpass filter of 1–40 Hz and a notch filter at 50 Hz. Independent component analysis was employed to identify and remove artifacts related to eye movements and heartbeats. Power spectrum density was utilized to eliminate bad channels.

### Microstate analysis

The spatial filter was utilized to determine and interpolate the maximum and minimum values from the six nearest neighboring sensors for each location. Subsequently, maps were extracted at the peaks of the global field power of the data, which corresponded to time frames exhibiting the highest signal-to-noise ratio [15, 23]. K-means clustering was then applied to the resultant collection of maps for each subject, followed by kneedle algorithm to estimate the optimal set of maps for the entire dataset at the group level [14, 15]. Grand-means clustering showed that 6 clusters had the highest global explained variance at 28.10%, exceeding both 4 clusters (25.66%) and 5 clusters (26.98%), consistent with the approximate 25%−40% reported for the magnetometer electrode [24]. Previous studies have shown that six types of resting-state MEG microstate maps are stable and repeatable [14, 25]. Brain functional state changes were assessed using four microstate parameters: duration (average stability time of a specific microstate in milliseconds), occurrence per second (average frequency per second of a given microstate), coverage (percentage of total recording time occupied by a specific microstate), and transition probability (proportion of transitions between microstate classes) [13].

#### Sources of microstates

The MRI images were segmented into scalp and brain tissues utilizing the FreeSurfer software package (http://surfer.nmr.mgh.harvard.edu/). The alignment of each subject’s brain tissue with sensor positions was accomplished through a rigid body transformation, facilitated by surface matching between the anatomical and optical images. The Automated Anatomical Labeling (AAL) atlas (version ROI_MNI_v4) was employed for precise mapping of the MEG data space for each subject [26]. Calculations were conducted using the Montreal Neurological Institute template, with regions of interest defined by AAL atlas. Individual brains were segmented into 78 cortical regions.

### Statistical analysis

Data analysis was performed using Statistical Product and Service Solutions version 27.0.1 (IBM, Chicago, IL, USA). The Shapiro-Wilk test was applied to assess the normality of continuous variables. Continuous variables were presented as mean ± standard deviation, with group differences in normally distributed variables analyzed via the independent samples t-test. Categorical variables were shown as counts and percentages, with differences assessed using Fisher’s Exact test.

Microstate parameter differences between groups were analyzed using a generalized linear model (GLM), adjusting for educational levels, HAMD-24, and HAMA-14 scores, with multiple comparison corrections via the False Discovery Rate (FDR). A quantitative sensitivity analysis was conducted to assess the impact of unmeasured confounding using the E-value and its lower control limit (LCL) [27, 28]. The E-value quantifies the minimum association strength an unmeasured confounder must have with both the exposure (migraineurs vs. healthy controls) and the outcome (MEG microstate parameter), conditional on the measured covariates, to fully explain away the observed association. E-values were calculated for each significant GLM result using an online calculator (https://www.evalue-calculator.com/). Larger E-values indicate greater robustness, as stronger unmeasured confounding is needed to nullify the effect. Multivariate logistic regression with FDR corrections served as complementary analysis to test if significant microstate parameters predicted group membership after covariate adjustment. Consistent findings between GLM and multivariate logistic regression analyses confirmed robustness across model specifications.

Pearson correlation coefficients were employed to investigate the association between microstate alterations and clinical characteristics. P values were presented without adjusting for multiple comparisons in the exploratory analysis.

A P-value of less than 0.05 was deemed statistically significant, and all statistical tests were conducted as two-tailed.

## Results

### Clinical characteristics of migraineurs and healthy controls

The present study included 25 migraine patients without aura (mean age = 32.64 ± 7.86 years, 21 females) and 18 healthy controls (mean age = 30.33 ± 9.22 years, 13 females). No significant differences in age or sex were observed between the two groups (*P* > 0.05). However, compared to healthy controls, migraineurs had significantly lower educational levels (*P* < 0.001), higher HAMD-24 scores (*P* = 0.002), and higher HAMA-14 scores (*P* < 0.001). Detailed demographic and clinical characteristics of all participants are presented in Table 1.

**Table 1:** Demographic and clinical characteristics of migraineurs and healthy controls

Characteristics	Migraineurs (*n* = 25)	Healthy controls (*n* = 18)	*P*-value
Age (years)	32.64 ± 7.86	30.33 ± 9.22	0.382
Sex (female)	21 (84.00%)	13 (72.22%)	0.267
Educational level (years)	16.04 ± 2.87	19.17 ± 1.89	**2.315 × 10** ^**−4**^
Duration of migraine (years)	9.48 ± 5.43	-	-
Frequency of attacks (times per month)	6.84 ± 7.53	-	-
Headache attack duration (hours)	10.70 ± 11.42	-	-
Accompanied symptoms with attacks			
Nausea	23 (92.00%)	-	-
Vomiting	14 (56.00%)	-	-
Phonophobia	13 (52.00%)	-	-
Photophobia	13 (52.00%)	-	-
Locus of headache			
Bilateral	8 (32.00%)	-	-
Unilateral	17 (68.00%)	-	-
Frontal	6 (24.00%)	-	-
Tempus	21 (84.00%)	-	-
Parietal	4 (16.00%)	-	-
Occipital	6 (24.00%)	-	-
VAS score	6.32 ± 0.98	-	-
HIT-6 score	65.88 ± 5.83	-	-
MIDAS score	17.36 ± 14.55	-	-
HAMD-24 score	9.08 ± 8.75	3.06 ± 1.56	**0.002**
HAMA-14 score	6.88 ± 5.16	2.67 ± 1.03	**4.820 × 10** ^**−4**^

Values are n (%) or mean ± standard deviation. Bold indicates statistically significant at *P* < 0.05

*VAS* Visual Analogue Scale, *HIT-6* Headache Impact Test-6, *MIDAS* Migraine Disability Assessment Score, *HAMD-24* 24-item Hamilton Rating Scale for Depression, *HAMA-14* 14-item Hamilton Rating Scale for Anxiety

### Microstate maps and source localization

**Fig. 1:**
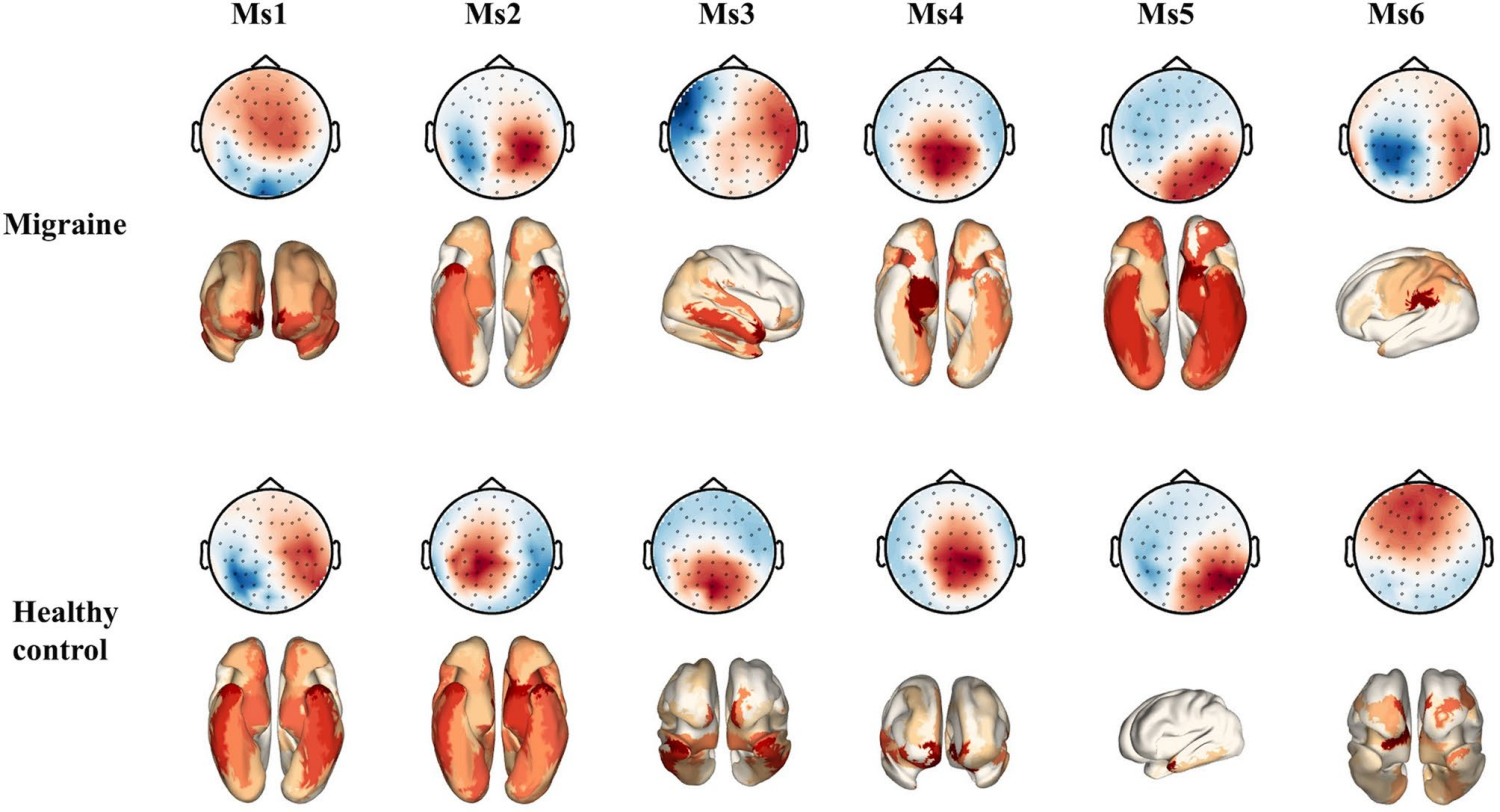
The six MEG microstates and their corresponding source-based neural activity in migraineurs and healthy controls. Peak activity levels are indicated in red

Utilizing K-means clustering, MEG data from migraineurs and healthy controls were categorized into six distinct microstates, labeled Ms1 to Ms6 (Fig. 1). These six microstates accounted for a substantial proportion of global explained variance in each group.

Source-based analyses revealed distinct neural activity patterns: migraineurs showed ventral fronto-temporal activity for Ms1 and Ms2, right inferior temporal gyrus activity for Ms3, right para-hippocampal activity for Ms4, inferior temporal gyrus activity for Ms5, and superior temporal gyrus activity for Ms6. In contrast, healthy controls exhibited temporal pole activity for Ms1 and Ms2, ventral fronto-temporal activity for Ms3 and Ms4, middle temporal gyrus activity for Ms5, and medial frontal gyrus activity for Ms6 (Fig. 1).

### Group differences in microstate parameters

**Fig. 2:**
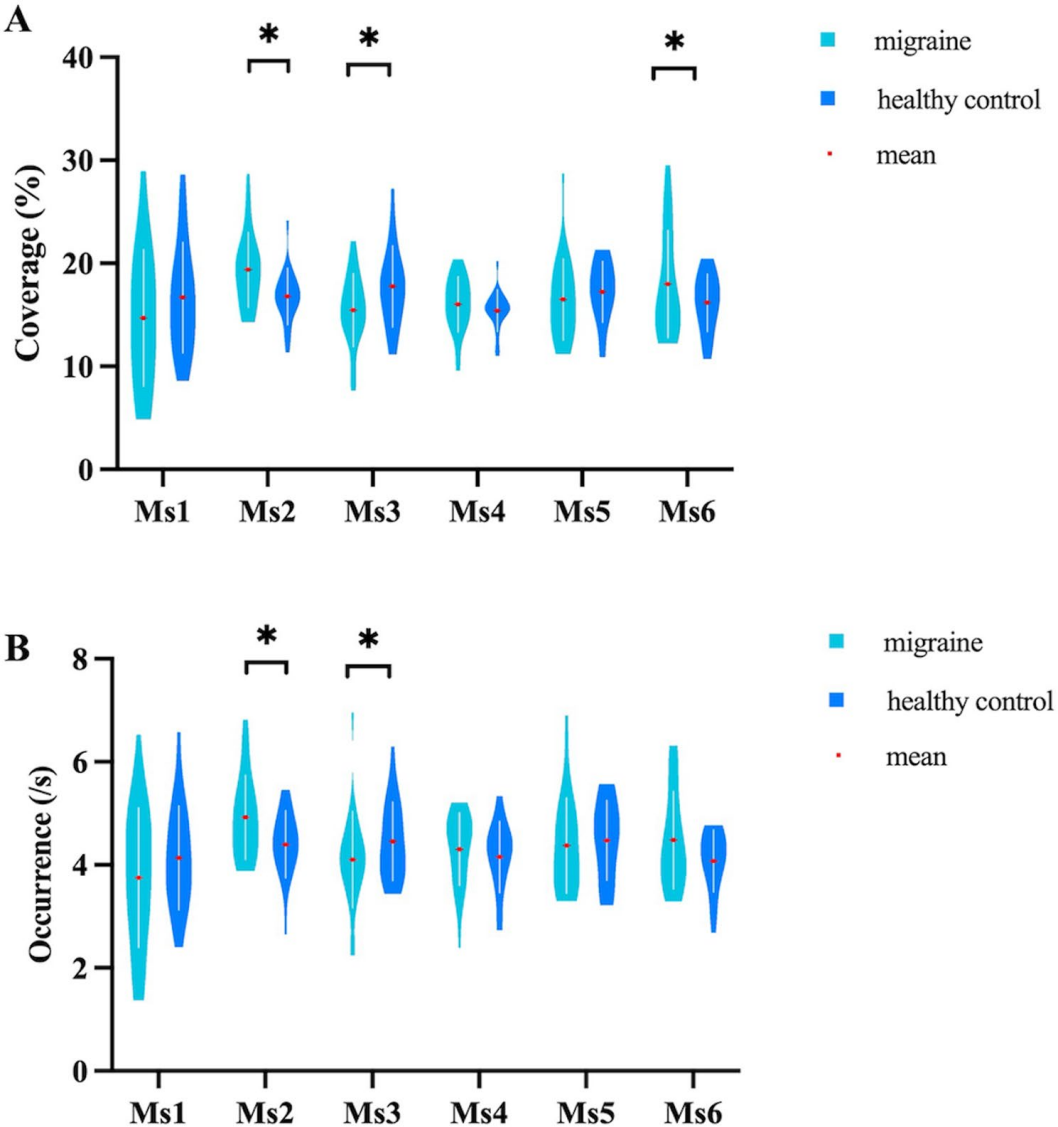
Comparison of temporal parameters of the six MEG microstates between the two groups. Coverage (**A**) and occurrence per second (**B**) of each microstate were analyzed using a generalized linear model, with educational levels, HAMD-24, and HAMA-14 scores included as continuous covariates. * indicates *P* < 0.05, false discovery rate corrected

After adjusting for educational levels, HAMD-24, and HAMA-14 scores, and applying FDR correction, migraineurs showed elevated coverage (19.37 ± 3.65 vs. 16.79 ± 2.78, pFDR = 0.038; E-value = 1.85, LCL: 1.11) and occurrence per second (4.92 ± 0.82 vs. 4.40 ± 0.66, pFDR = 0.042; E-value = 3.24, LCL: 1.36) of Ms2 and increased coverage of Ms6 (17.98 ± 5.23 vs. 16.17 ± 2.82, pFDR = 0.039; E-value = 1.57, LCL: 1.09), while coverage (15.45 ± 3.56 vs. 17.76 ± 3.97, pFDR = 0.033; E-value = 1.83, LCL:1.24) and occurrence per second (4.10 ± 0.94 vs. 4.46 ± 0.77, pFDR = 0.042; E-value = 7.19, LCL: 1.96) of Ms3 were decreased compared to healthy controls (Fig. 2).

**Fig. 3:**
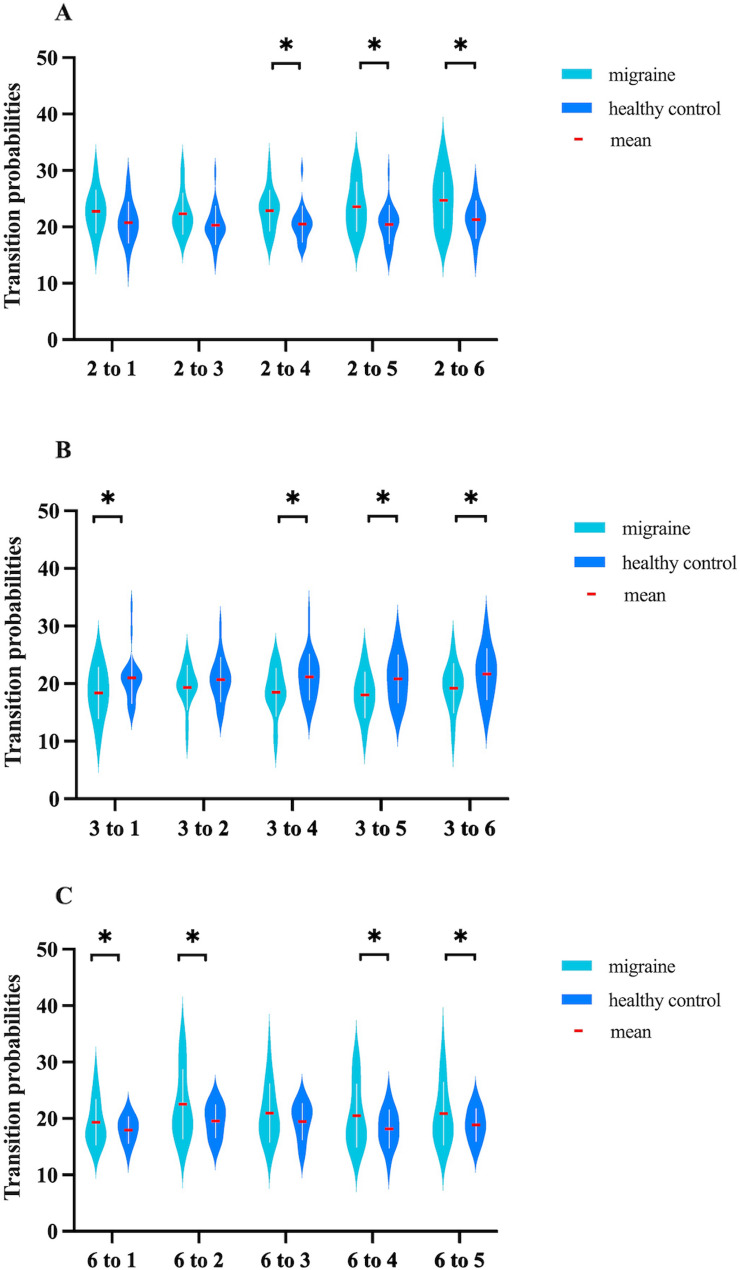
Comparison of transition probabilities among the six MEG microstates between the two groups. Transition probabilities from Ms2 (**A**), Ms3 (**B**), and Ms6 (**C**) to other microstates were calculated using Markov chains and analyzed with a generalized linear model, controlling for educational levels, HAMD-24, and HAMA-14 scores as continuous covariates. * indicates *P* < 0.05, false discovery rate corrected

Transition probabilities from Ms2 to Ms4 (22.90 ± 3.62 vs. 20.53 ± 3.21, pFDR = 0.034; E-value = 1.85, LCL: 1.15), Ms5 (23.60 ± 4.38 vs. 20.45 ± 3.41, pFDR = 0.027; E-value = 1.75, LCL: 1.15), and Ms6 (24.72 ± 4.95 vs. 21.31 ± 3.33, pFDR = 0.023; E-value = 1.93, LCL: 1.27), and from Ms6 to Ms1 (19.34 ± 4.05 vs. 17.97 ± 2.37, pFDR = 0.048; E-value = 1.68, LCL: 1.07), Ms2 (22.56 ± 6.18 vs. 19.55 ± 2.94, pFDR = 0.045; E-value = 1.60, LCL: 1.13), Ms4 (20.54 ± 5.60 vs. 18.17 ± 3.38, pFDR = 0.026; E-value = 1.70, LCL: 1.14), and Ms5 (20.90 ± 5.59 vs. 18.88 ± 2.89, pFDR = 0.038; E-value = 1.56, LCL: 1.08) were higher in migraineurs, whereas transition probabilities from Ms3 to Ms1 (18.39 ± 4.46 vs. 21.02 ± 4.44, pFDR = 0.043; E-value = 1.81, LCL: 1.22), Ms4 (18.51 ± 4.17 vs. 21.18 ± 3.98, pFDR = 0.031; E-value = 1.81, LCL: 1.25), Ms5 (18.06 ± 3.96 vs. 20.84 ± 4.17, pFDR = 0.030; E-value = 1.84, LCL: 1.21), and Ms6 (19.23 ± 4.28 vs. 21.67 ± 4.44, pFDR = 0.036; E-value = 1.64, LCL: 1.16) were lower (Fig. 3). Other microstate parameters showed no significant differences.

Multivariate logistic regression results, adjusted for educational levels, HAMD-24, and HAMA-14 scores with FDR correction, largely aligned with GLM findings (Supplementary Tables 1 and Table 2).

### Correlation between microstate parameters and clinical measures

**Fig. 4:**
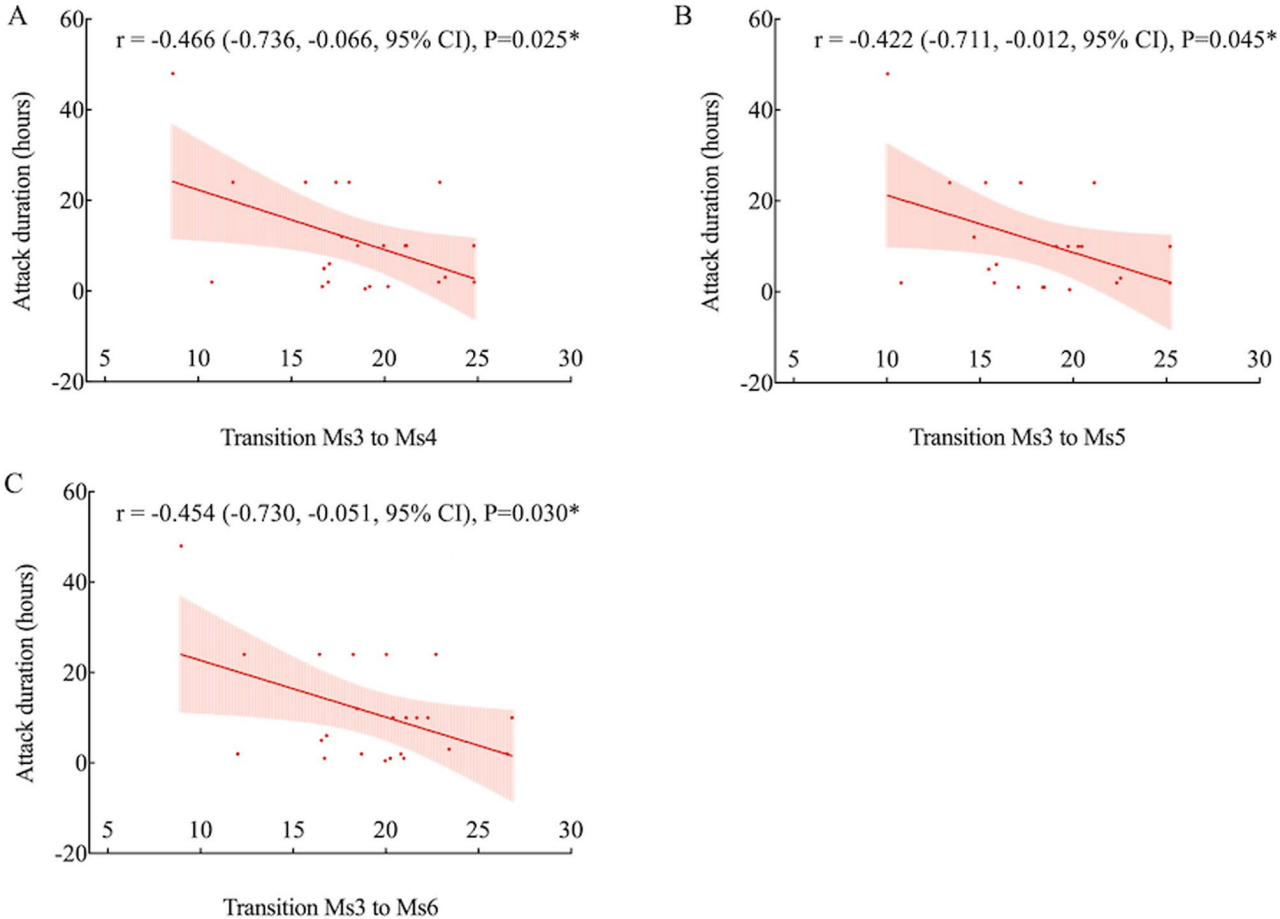
Correlation between headache attack duration and transition probabilities in migraineurs. Specifically, the transition probabilities from Ms3 to Ms4 (**A**), Ms5 (**B**), and Ms6 (**C**) are shown. *P < 0.05, uncorrected

**Fig. 5:**
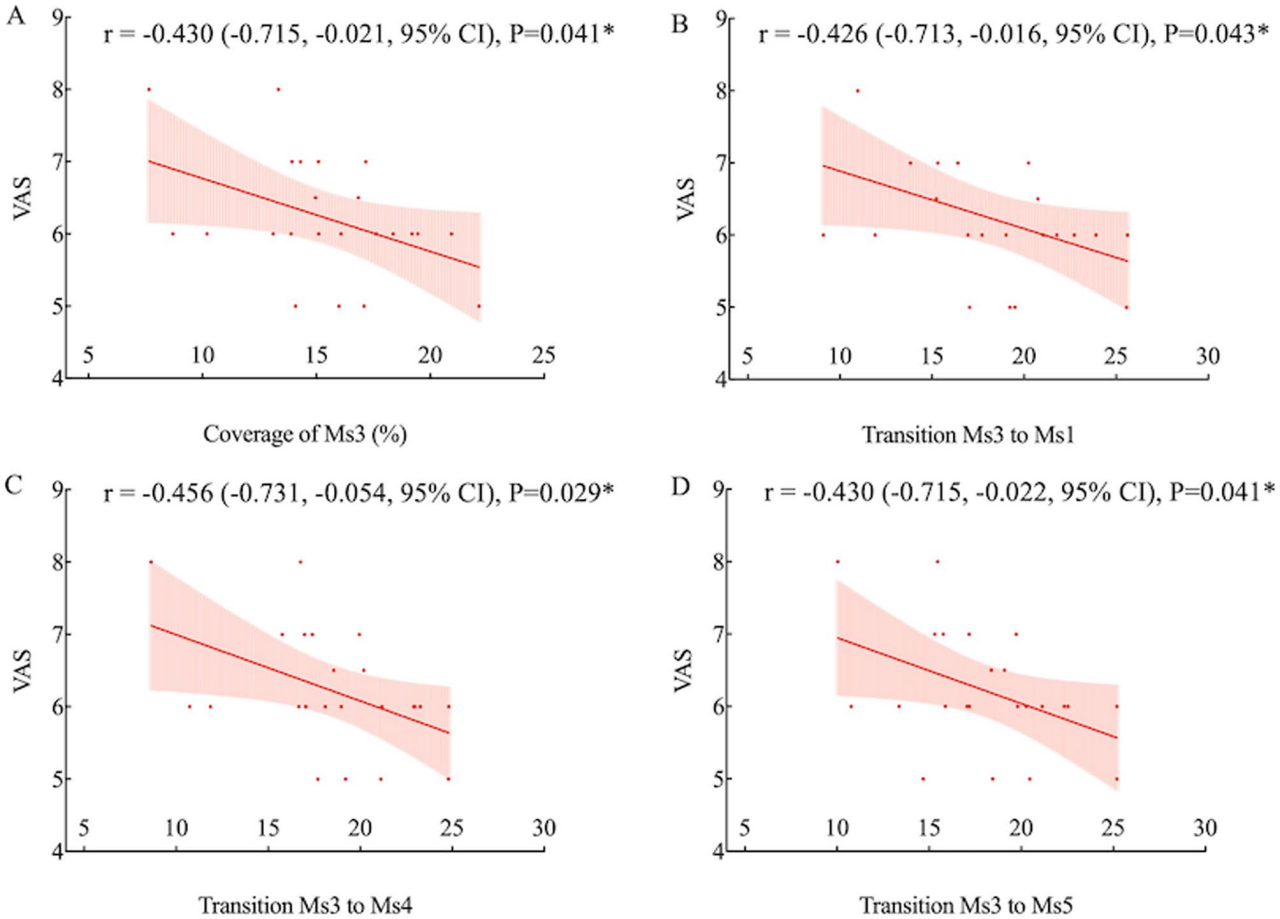
Correlation between MEG microstate parameters and VAS scores in migraineurs. The relationships between VAS scores and coverage of Ms3 (**A**), and transition probabilities from Ms3 to Ms1 (**B**), Ms4 (**C**), and Ms5 (**D**) are presented. *P < 0.05, uncorrected

**Fig. 6:**
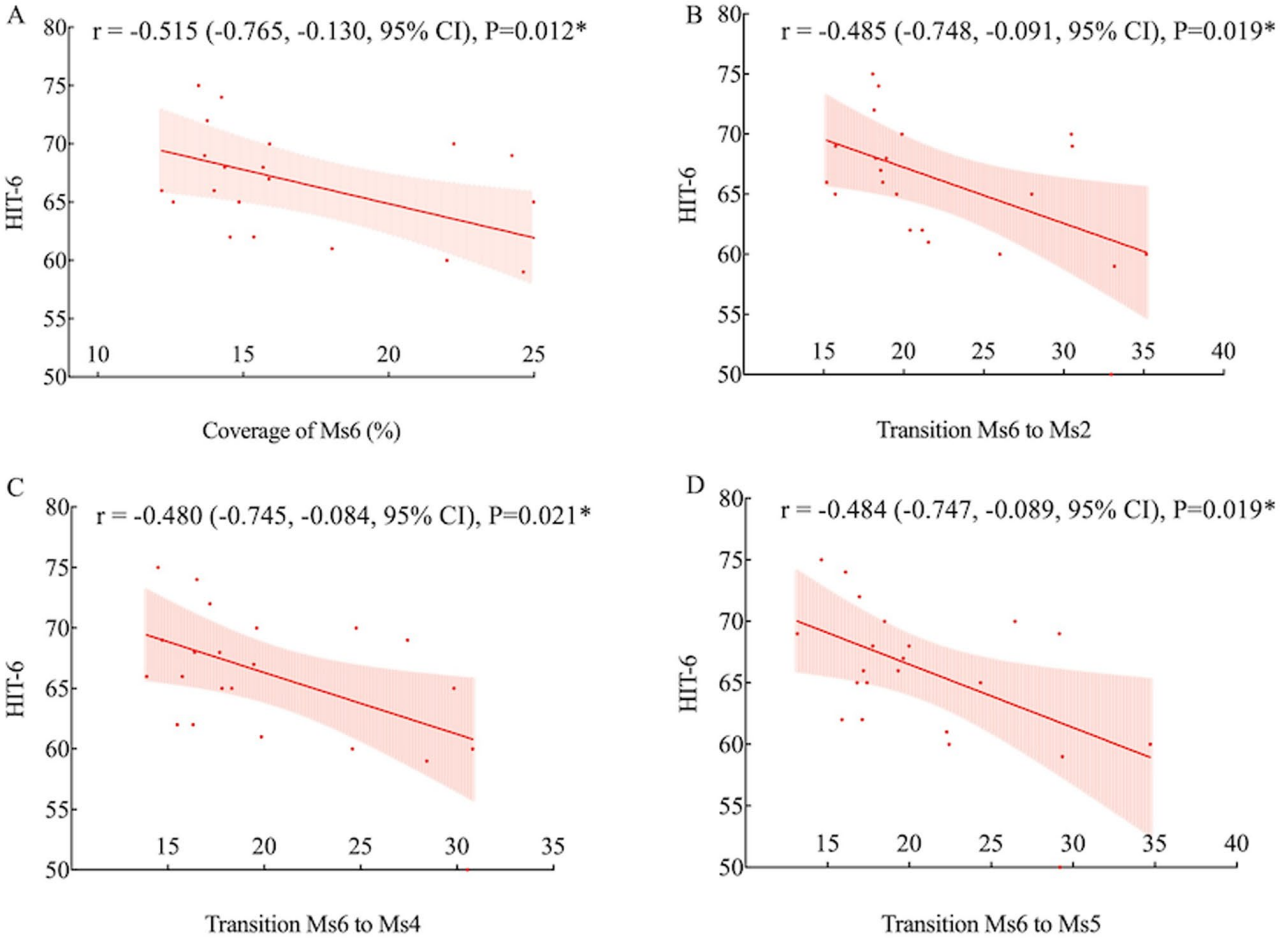
Correlation between MEG microstate parameters and HIT-6 scores in migraineurs. The relationships between HIT-6 scores and coverage of Ms6 (**A**), and transition probabilities from Ms6 to Ms2 (**B**), Ms4 (**C**), and Ms5 (**D**) are illustrated. *P < 0.05, uncorrected

Headache attack duration showed significant negative correlations with transition probabilities from Ms3 to Ms4 (*r* = −0.466, *P* = 0.025), Ms5 (*r* = −0.422, *P* = 0.045), and Ms6 (*r* = −0.454, *P* = 0.030) (Fig. 4). Headache intensity (VAS score) was negatively correlated with Ms3 coverage (*r* = −0.430, *P* = 0.041) and transition probabilities from Ms3 to Ms1 (*r* = −0.426, *P* = 0.043), Ms4 (*r* = −0.456, *P* = 0.029), and Ms5 (*r* = −0.430, *P* = 0.041) (Fig. 5). HIT-6 score was negatively correlated with Ms6 coverage (*r* = −0.515, *P* = 0.012) and transition probabilities from Ms6 to Ms2 (*r* = −0.485, *P* = 0.019), Ms4 (*r* = −0.480, *P* = 0.021), and Ms5 (*r* = −0.484, *P* = 0.019) (Fig. 6). Other microstate parameters did not correlate significantly with clinical characteristics.

## Discussion

This study compared OPM-MEG microstates between migraineurs and healthy controls, revealing significant differences. Migraineurs exhibited elevated coverage and occurrence per second of Ms2 and increased coverage of Ms6, whereas these parameters of Ms3 were reduced. Transition probabilities among microstates (especially Ms2, Ms3, and Ms6) also differed between groups. Coverage and transition probabilities of Ms3 and Ms6 were negatively correlated with headache attack duration, intensity, and HIT-6 scores among migraineurs.

Our study found that migraineurs showed increased coverage and occurrence per second of Ms2 and elevated coverage of Ms6, while these parameters of Ms3 were reduced relative to healthy controls. These microstate parameters reflect the activation tendency of underlying cortical and subcortical sources and the relative temporal coverage of such neural activities [29]. In MEG data, Ms2 indicated heightened activity in several right hemisphere networks, Ms3 in bilateral Salience/Ventral Attention B networks, and Ms6 in various right hemisphere networks [14]. Our results suggested abnormal functioning in the dorsal attention, temporal-parietal, default mode, somato-motor, control, visual, and salience networks in migraine patients without aura, supporting multimodal MRI and multi-frequency M/EEG findings [30–33]. Migraineurs processed sensory information from visual, auditory, and somatosensory inputs alongside recurring pain, affecting brain areas related to attention, sensitivity, and emotions [10]. Prior research indicated that MEG microstates 2–3 and EEG microstates B-C shared some overlap in global explained variance and brain sources [14]. This study aligns with previous EEG research showing increased coverage and occurrence per second of microstates B-C in migraineurs compared to healthy controls [11]. However, our findings align only partially with earlier EEG studies in migraineurs, which reported increased coverage and occurrence for microstate B but no significant group differences for microstate C [10, 12, 13]. This discrepancy may result from differences in sample size, recording techniques, scanning times, and data analysis methods.

In this study, source-based analyses showed unusual activity in migraineurs: ventral fronto-temporal for Ms2, right inferior temporal gyrus for Ms3, and superior temporal gyrus for Ms6. In migraine, the superior and inferior temporal gyri were involved in auditory and visual processing and multisensory integration, while fronto-temporal regions were associated with emotion regulation [34, 35]. Our findings indicated that abnormal activity in the temporal and fronto-temporal regions might be related to accompanied symptoms, including phonophobia, photophobia, and mood changes. This also supports widespread dysfunction in multisensory integration and emotion processing among migraineurs, as shown by fMRI and M/EEG findings [33–35].

Transition probabilities from Ms3 to Ms1, Ms4, Ms5, and Ms6 were lower in migraineurs, whereas transition probabilities from Ms2 to Ms4, Ms5, and Ms6, and from Ms6 to Ms1, Ms2, Ms4, and Ms5 were higher. Similar to Ms3 and Ms6, Ms4 showed increased activity in the bilateral Somato-Motor A, Dorsal Attention B, and Control C networks, while Ms5 had heightened activity in left hemisphere networks in MEG recordings [14]. Our findings indicated that the salience/ventral attention network (SN/VAN) was less likely to transition to other major networks but more likely to receive transitions from them. Although the SN could detect stimuli, it struggled to alert other systems, causing sensory overload and hypersensitivity (like photophobia and phonophobia) [36]. Limited transitions from the SN to the default mode network may impede shifting from rest to active threat response. Similarly, fewer transitions to the dorsal attention and control networks suggested difficulties in focusing and cognitive control during threat detection, contributing to cognitive issues in migraines [37]. This pointed to abnormal global brain dynamics, with the brain frequently entering a metabolically costly “threat monitoring” state, potentially leading to interictal fatigue and anxiety. Increased transitions to the SN suggested that neutral sensory inputs might be negatively perceived, reducing the attack threshold and promoting migraine chronification. The dysfunctional dynamic of being both “isolated yet dominant” was potentially associated with the sensory processing abnormalities, cognitive deficits, and the transition from episodic to chronic migraine. Abnormal transition probabilities in migraineurs might explain synergy disorders across brain networks and potentially serve as biomarkers for migraine diagnosis and treatment. No significant differences in transition probabilities were found between Ms2 and Ms3 in migraineurs and healthy controls, consistent with one EEG study [12] but not others [10, 11, 13], possibly due to methodological differences.

In migraineurs, coverage and transition probabilities of Ms3 and Ms6 were negatively correlated with headache attack duration, intensity, and HIT-6 scores, which measure impact of headaches on various life aspects. Microstate parameters, reflecting large-scale brain networks, may highlight disruptions explaining patients’ dysfunctional behaviors [29]. Our findings support that decreased functional connectivity in cortical networks was linked to increased migraine frequency, severity, and headache-related disabilities in multimodal MRI studies [38, 39]. This aligns with previous EEG research showing a negative correlation between the mean duration of microstate C and HIT-6 scores in migraine patients without aura [11]. Our results from the correlation analysis were preliminary findings that required confirmation through future studies with larger samples.

OPM-MEG surpasses EEG in migraine research due to its superior spatial precision, motion robustness, and wearability suitable for studying ictal phases. Its enhanced sensitivity to high-frequency oscillations enables detailed network analysis. Clinically, OPM-MEG offers improved localization of deep sources (e.g., temporal lobe, insula, anterior cingulate cortex, amygdala) implicated in migraine pathophysiology [16, 18]. Unlike EEG, which cannot detect cortical spreading depression (CSD), OPM-MEG may localize CSD by directly measuring neuronal activity independent of neurovascular coupling [6]. Thus, OPM-MEG holds greater potential for uncovering migraine pathophysiology and identifying biomarkers.

This study presents several limitations that warrant careful consideration. Firstly, it focused only on patients with migraine without aura and had a relatively small sample size, which may affect statistical power and result generalizability. Secondly, despite correcting for baseline imbalances in the GLM, some results had an LCL of E-value near 1.1, suggesting potential unobserved confounding factors. While the multivariate logistic regression results largely align with the GLM, larger studies with more detailed data are necessary. Finally, the study lacked a longitudinal follow-up to assess the link between MEG microstate parameters and migraine prognosis, highlighting the need for large prospective cohort studies.

## Conclusion

The study found abnormal parameters in OPM-MEG microstate classes 2, 3, and 6 and their correlations with clinical characteristics in migraineurs, with a relatively small sample size. These findings provided evidence for a pathological basis of the synergistic dysfunction across brain networks in migraine. OPM-MEG microstate parameters might serve as potential biomarkers for migraine diagnosis and treatment. Further research with larger, prospective cohorts is necessary to validate these findings.

## Supplementary Information

Supplementary Material 1.

## Data Availability

The datasets of this current study are not available to public. Anonymous data can be obtained from the corresponding author upon appropriate request.

## Abbreviations

fMRI: Functional magnetic resonance imaging
EEG: Electroencephalogram
MEG: Magnetoencephalography
OPM-MEG: Optically pumped magnetometer magnetoencephalography
SQUID-MEG: Superconducting Quantum Interference Device MEG
VAS: Visual analogue scale
ICHD-III: International classification of headache disorders, 3rd edition
HIT-6: Headache Impact Test-6
MIDAS: Migraine disability assessment score
HAMA-14: 14-item Hamilton Rating Scale for Anxiety
HAMD-24: 24-item Hamilton Rating Scale for Depression
AAL: Automated anatomical labeling
GLM: Generalized linear model
FDR: False discovery rate
LCL: Lower control limit
SN/VAN: Salience/ventral attention network
CSD: Cortical spreading depression
