# Supplementary material for: Differential resting-state MEG microstate patterns in migraineurs: a cross-sectional study
Source: J Headache Pain. 2025 Oct 14;26(1):215. doi: 10.1186/s10194-025-02168-z (PMC12523030; doi:10.1186/s10194-025-02168-z)
Supplement: Supplementary file 1 — Supplementary Material 1. [file 10194_2025_2168_MOESM1_ESM.docx]

**Supplementary Table 1** Multivariate logistic regression assessed the association between microstate parameters and migraine.

| **Microstate parameters** | **OR** | **95% CI** | **pFDR** |
| --- | --- | --- | --- |
| Mean duration (ms) | 1.080 | 0.864-1.350 | 0.225 |
| Duration (ms) |  |  |  |
| Ms1 | 1.020 | 0.878-1.186 | 0.317 |
| Ms2 | 1.163 | 0.929-1.455 | 0.110 |
| Ms3 | 0.954 | 0.794-1.147 | 0.271 |
| Ms4 | 1.109 | 0.857-1.436 | 0.202 |
| Ms5 | 1.040 | 0.845-1.280 | 0.297 |
| Ms6 | 1.085 | 0.901-1.307 | 0.190 |
| Coverage (%) |  |  |  |
| Ms1 | 0.878 | 0.740-1.042 | 0.094 |
| Ms2 | 1.603 | 1.021-2.515 | **0.044*** |
| Ms3 | 0.629 | 0.427-0.928 | **0.044*** |
| Ms4 | 1.235 | 0.846-1.801 | 0.145 |
| Ms5 | 0.953 | 0.725-1.252 | 0.298 |
| Ms6 | 1.324 | 1.015-1.727 | **0.044*** |
| Occurrence (/s) |  |  |  |
| Ms1 | 0.455 | 0.177-1.171 | 0.081 |
| Ms2 | 3.661 | 1.159-11.560 | **0.044*** |
| Ms3 | 0.067 | 0.008-0.577 | **0.044*** |
| Ms4 | 1.256 | 0.386-4.092 | 0.297 |
| Ms5 | 0.658 | 0.228-1.896 | 0.202 |
| Ms6 | 3.860 | 0.827-18.015 | 0.074 |

Multivariate logistic regression was performed with educational levels, HAMD-24, and HAMA-14 scores as continuous covariates; * indicates pFDR < 0.05 after false discovery rate correction.

**Supplementary Table 2** Multivariate logistic regression assessed the association between transition probabilities and migraine.

| **Transition probabilities** | **OR** | **95% CI** | **pFDR** |
| --- | --- | --- | --- |
| Ms1 to Ms2 | 0.901 | 0.772-1.051 | 0.110 |
| Ms1 to Ms3 | 0.873 | 0.741-1.028 | 0.081 |
| Ms1 to Ms4 | 0.876 | 0.738-1.040 | 0.094 |
| Ms1 to Ms5 | 0.856 | 0.719-1.020 | 0.074 |
| Ms1 to Ms6 | 0.866 | 0.719-1.043 | 0.094 |
| Ms2 to Ms1 | 1.197 | 0.915-1.568 | 0.110 |
| Ms2 to Ms3 | 1.243 | 0.921-1.678 | 0.103 |
| Ms2 to Ms4 | 1.601 | 1.035-2.477 | **0.044*** |
| Ms2 to Ms5 | 1.497 | 1.036-2.162 | **0.044*** |
| Ms2 to Ms6 | 1.701 | 1.095-2.643 | **0.044*** |
| Ms3 to Ms1 | 0.639 | 0.436-0.937 | **0.044*** |
| Ms3 to Ms2 | 0.682 | 0.485-0.957 | **0.044*** |
| Ms3 to Ms4 | 0.639 | 0.444-0.921 | **0.044*** |
| Ms3 to Ms5 | 0.627 | 0.419-0.939 | **0.044*** |
| Ms3 to Ms6 | 0.719 | 0.537-0.962 | **0.044*** |
| Ms4 to Ms1 | 1.226 | 0.870-1.728 | 0.134 |
| Ms4 to Ms2 | 1.260 | 0.908-1.748 | 0.107 |
| Ms4 to Ms3 | 1.026 | 0.767-1.372 | 0.331 |
| Ms4 to Ms5 | 1.145 | 0.866-1.513 | 0.173 |
| Ms4 to Ms6 | 1.451 | 1.035-2.034 | **0.044*** |
| Ms5 to Ms1 | 0.892 | 0.705-1.130 | 0.173 |
| Ms5 to Ms2 | 1.003 | 0.784-1.282 | 0.361 |
| Ms5 to Ms3 | 0.840 | 0.644-1.095 | 0.111 |
| Ms5 to Ms4 | 1.013 | 0.780-1.315 | 0.354 |
| Ms5 to Ms6 | 0.993 | 0.786-1.254 | 0.357 |
| Ms6 to Ms1 | 1.427 | 1.008-2.018 | **0.045*** |
| Ms6 to Ms2 | 1.352 | 1.028-1.779 | **0.044*** |
| Ms6 to Ms3 | 1.268 | 0.989-1.626 | 0.058 |
| Ms6 to Ms4 | 1.448 | 1.029-2.037 | **0.044*** |
| Ms6 to Ms5 | 1.316 | 1.010-1.716 | **0.044*** |

Multivariate logistic regression was performed with educational levels, HAMD-24, and HAMA-14 scores as continuous covariates; * indicates pFDR < 0.05 after false discovery rate correction.
